# Gait-Assist Wearable Robot Using Interactive Rhythmic Stimulation to the Upper Limbs

**DOI:** 10.3389/frobt.2019.00025

**Published:** 2019-04-24

**Authors:** Robin Miao Sin Yap, Ken-ichiro Ogawa, Yuki Hirobe, Terumasa Nagashima, Masatoshi Seki, Masayuki Nakayama, Ken Ichiryu, Yoshihiro Miyake

**Affiliations:** ^1^Department of Computational Intelligence and Systems Science, Tokyo Institute of Technology, Yokohama, Japan; ^2^Department of Computer Science, Tokyo Institute of Technology, Yokohama, Japan; ^3^Kikuchi Seisakusho Co. Ltd., Hachioji, Japan

**Keywords:** gait-assist wearable robot, interactive rhythmic stimulation, mutual entrainment, upper limbs, hip-swing amplitude, hip-swing period

## Abstract

Many power-assist wearable exoskeletons have been developed to provide walking support and gait rehabilitation for elderly subjects and gait-disorder patients. Most designers have focused on a direct power-assist to the wearer's lower limbs. However, gait is a coordinated rhythmic movement of four limbs controlled intrinsically by central pattern generators, with the upper limbs playing an important role in walking. Maintaining a normal gait can become difficult as a person ages, because of decreases in limb coordination, stride length, and gait speed. It is known that coordination mechanisms can be governed by the principle of mutual entrainment, in which synchronization develops through the interaction between nonlinear phase oscillators in biological systems. This principle led us to hypothesize that interactive rhythmic stimulation to upper-limb movements might compensate for the age-related decline in coordination, thereby improving the gait in the elderly. To investigate this hypothesis, we developed a gait-assist wearable exoskeleton that employs interactive rhythmic stimulation to the upper limbs. In particular, we investigated the effects on spatial (i.e., hip-swing amplitude) and temporal (i.e., hip-swing period) gait parameters by conducting walking experiments with 12 healthy elderly subjects under one control condition and five upper-limb-assist conditions, where the output motor torque was applied at five different upper-limb swing positions. The results showed a statistically significant increase in the mean hip-swing amplitude, with a mean increment of about 7% between the control and upper-limb-assist conditions. They also showed a statistically significant decrease in the mean hip-swing period, with a mean decrement of about 2.3% between the control and one of the upper-limb-assist conditions. Although the increase in the hip-swing amplitude and the decrease in the hip-swing period were both small, the results indicate the possibility that interactive rhythmic stimulation to the upper limbs might have a positive effect on the gait of the elderly.

## Introduction

In recent years, many power-assist wearable exoskeletons have been developed to provide walking support and gait rehabilitation for elderly subjects (Kawamoto et al., 2003; Deng et al., 2017; Choi et al., 2018) and gait-disorder patients (Riener et al., 2005; Veneman et al., 2007; Kim et al., 2010; Strausser and Kazerooni, 2011; Barbareschi et al., 2015; Bortole et al., 2015; Chen et al., 2017; Lerner et al., 2017). Most of these exoskeletons provide direct power-assist support to the wearer's lower limbs.

For example, the hybrid assistive leg, HAL (Tsukuba, Japan) uses the patient's intention-based electromyography signals to provide power-assist support to the lower limbs of patients with a gait disorder (Kawamoto et al., 2003). For patients with neurological disorders, the robotic-gait orthosis, Lokomat (Hacoma AG, Switzerland) provides task-oriented repetitive movement using a patient-cooperative strategy based on impedance, adaptive control, and visual biofeedback to control the hip and knee joint trajectories on a treadmill (Riener et al., 2005). The lower-extremity powered exoskeleton, LOPES (EA Enschede, The Netherlands) offers both patient-in-charge and robot-in-charge modes to control the patient's hip and knee trajectories on a treadmill (Veneman et al., 2007). The active leg exoskeleton, ALEX (Delaware, USA) uses a force-field controller and visual guidance on a treadmill to control hip and knee joint trajectories (Kim et al., 2010).

More recently, the lower limb robotic exoskeletons, REX (Florida, USA) (Barbareschi et al., 2015), H2 (Technaid S.L., Spain) (Bortole et al., 2015), active power-assist lower limb, APAL (Harbin, China) (Deng et al., 2017), CUHK-EXO (Hongkong, China) (Chen et al., 2017), novel powered lower-extremity exoskeleton (Maryland, USA) (Lerner et al., 2017), and gait-enhancing mechatronic system, GEMS (Samsung, Korea) (Choi et al., 2018) have been developed. The REX exoskeleton uses a touch-panel graphical user interface for the patient in providing an output torque to the lower limbs to achieve target performance. The H2 robotic exoskeleton provides output torque to the patient's lower limbs using a force-field controller and improvement in the patient's walking functions has been reported. The APAL exoskeleton provides output torque to the wearer's lower limbs from the torso posture and joint angles obtained from the inertial measurement units (IMU) to enable the user to walk on rough ground, climb stairs, and cross obstacles. The CUHK-EXO exoskeleton provides assistive torque to the patient's lower limbs using position control and was able to support the patient's stand up/sit down and walking motions. The novel powered lower-extremity exoskeleton provides output torque to the patient's knee joint using a proportional-integral-derivative control algorithm and an improvement in the hip and knee joint angle was reported. The GEMS exoskeleton assists elderly patients with walking difficulty by providing an output torque to the hip using particularly shaped adaptive oscillators.

Although many power-assist wearable exoskeletons have been developed, these exoskeletons aim to provide direct torque to the lower limbs to assist the patients' gait. However, gait is a coordinated movement of four limbs intrinsically controlled by central pattern generators, with the upper limbs playing an important role in walking (Zehr et al., 2001; Zehr and Haridas, 2003; Frigon et al., 2004; Zehr and Duysens, 2004). It is therefore reasonable to consider intervening in the upper-limb movements to support the lower-limb movements. For example, the medical exoskeleton, eLEGS (California, USA) applies an inertial measurement unit to the upper limbs of patients with spinal cord injuries to assist the patient to stand up, walk and sit down independently, based on intention signals from the patient (Strausser and Kazerooni, 2011). However, eLEGS simply applies inertial measurements to the patients' upper limbs to correct their gait trajectories without considering fully the global dynamics of upper- and lower-limb coordination in human locomotion (Delwaide et al., 1977; Baldissera et al., 1982, 1988; Guadagnoli et al., 2000; Dietz et al., 2001; Huang and Ferris, 2004, 2009; Kawashima et al., 2008).

To investigate this upper-limb intervention hypothesis, we have developed a gait-assist system called the WalkMate (Tokyo, Japan). This system provides subjects with auditory stimulation as an interactive rhythmic stimulation to establish synchronization between the stimulation and their walking footsteps. The stimulation is generated by the interaction between the artificial nonlinear phase oscillators and the subject's walking footsteps and is based on the principle of mutual entrainment (Miyake, 2009). This principle explains biological-organization phenomena in which a set of nonlinear phase oscillators become synchronized by interaction via appropriate coupling (Kuramoto, 1984). Such biological phenomena have been observed in many internal parts of living organisms, such as segmental oscillators in the lamprey spinal generator (Cohen et al., 1982), swimming patterns of aquatic animals (Yuasa and Ito, 1990), and the coordinated movements of bipedal human locomotion (Taga et al., 1991). Therefore, the WalkMate enables us to test the hypothesis that the upper limbs' movement can affect the lower limbs' movement by applying interactive rhythmic stimulation to the upper limbs from the interaction between upper limbs and lower limbs. There is then the possibility that applying appropriate interactive rhythmic stimulation to the subjects' upper limbs could improve their lower limb gait patterns.

In this study, we aimed to implement the WalkMate as a wearable exoskeleton that could provide interactive rhythmic stimulation to the upper limbs. Then, to investigate our hypothesis, we measured the effect of interactive rhythmic stimulation to the upper limbs on spatial (i.e., hip-swing amplitude) and temporal (i.e., hip-swing period) gait parameters. Our experiments involved overground walking by healthy elderly subjects under a baseline control condition and five upper-limb-assist conditions with different motor-torque outing timings. Here, the timings correspond to the onset of an output motor torque at different arm-swing positions of the upper limbs.

## Materials and Methods

### Gait-Assist Wearable Exoskeleton

The WalkMate gait-assist wearable exoskeleton comprises (1) hardware to provide motor torque (rhythmic stimulation) to the left and right upper limbs of a subject, triggered by rhythmic signals, and (2) software to generate the rhythmic signals in synchronization with the walking rhythm of the subject based on the mutual entrainment principle.

### Hardware

**Figure 1:**
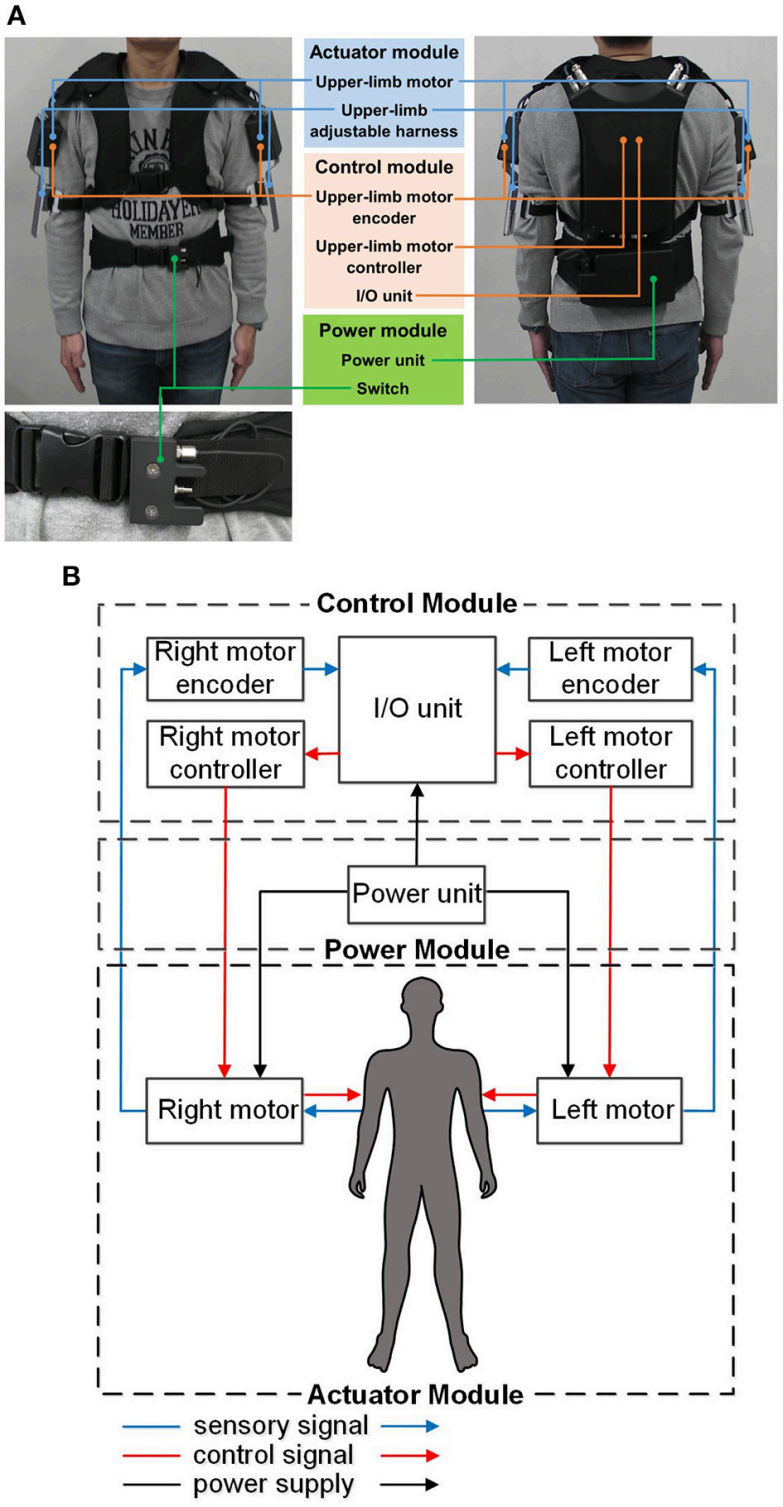
Overview of the gait-assist wearable exoskeleton: **(A)** hardware appearance, **(B)** schematic diagram of the hardware.

The hardware comprises an actuator module, a control module, and a power module. Its overall weight is 5.8 kg. The actuator module is rigidly attached to an adjustable harness that can be secured to the upper limbs (between the elbow and shoulder joint) using a Velcro belt. The wearable exoskeleton is rigidly secured to the upper body (i.e., chest) and lower body (i.e., waist) of the subject using adjustable belts. Figures 1A,B show the appearance and schematic diagram of the hardware module of the wearable exoskeleton, respectively.

### Actuator Module

This module comprises two three-phase DC brushless motors (DR-4316-X14B00420; Shinano Kenshi, Nagano, Japan), each of which provides output torque to the upper limbs, adjustable harnesses, and a Velcro belt. The motors have a drive voltage of 24.0 V, a rated load current of 2.0 A, and a maximum thrust load of 3.8 N.

### Control Module

This module comprises two upper-limb DC motor controllers, two upper-limb DC motor encoders, and an I/O unit. The encoders provide the control interface between the upper limbs and the I/O unit by detecting the subject's shoulder-joint angular displacement and sending this information to the I/O unit. The controllers provide the control interface between the I/O unit and the upper-limb DC motors by causing the motors to output the desired torque to the subject's upper limbs. An Android smartphone (ASUS Z00ED; Zenfone, Taiwan) controls the magnitude and phase of the output motor torque to the upper limbs though wireless communication with the I/O unit via Bluetooth.

### Power Module

This module comprises an external power unit (7LPP545483AHR-1M01-WS; Hitachi, Japan) and an external on/off switch strapped to the adjustable belt at the waist level. The rechargeable external power unit provides power to the control module and drives the upper-limb DC motors. The external on/off switch acts as an emergency switch to enable the subject to override the human operator by switching off the motor torque to the upper limbs in case of an emergency or discomfort.

### Software

**Figure 2:**
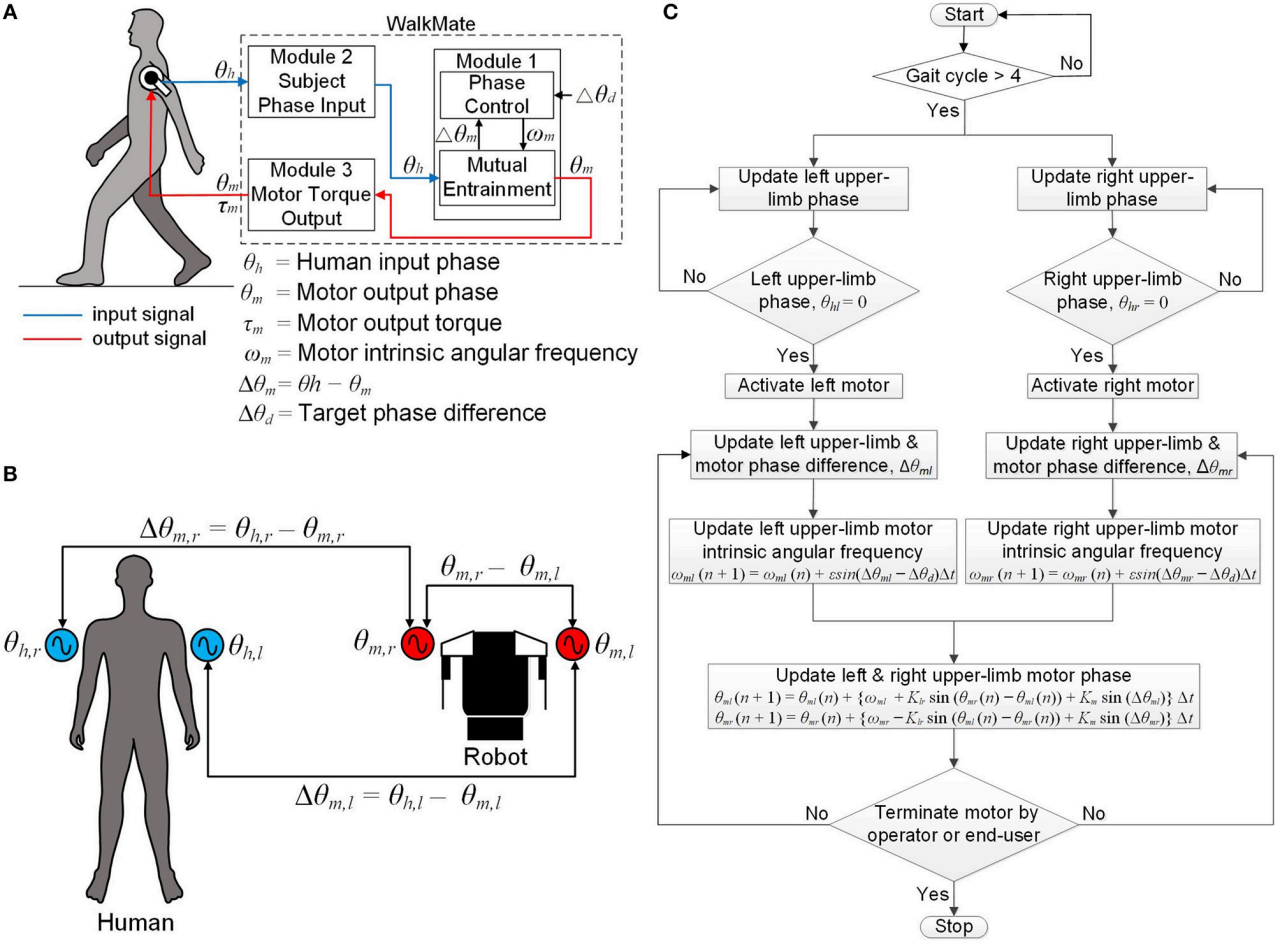
Schematic diagram of the software: **(A)** schematic diagram of the software modules, **(B)** definition of all phases and phase differences used in the phase control module, **(C)** flowchart of the software control algorithm.

The software comprises three modules. Module 1 (phase control module) controls the left and right upper-limb motor phases (i.e., their timing) by coordinating the phase differences of the left and right upper limbs and the upper-limb motors to a target value based on the mutual entrainment principle. Module 2 (subject phase-input module) receives both of the subject's upper-limb angular displacements in real time via the upper-limb motor encoders. Finally, Module 3 (motor-torque output module) controls the magnitude and phase for each of the upper-limb output motor torques and outputs the desired magnitude and phase information to the subject. Figure 2A shows a schematic diagram of the software for the WalkMate.

### Phase Control Module

This module comprises the mutual entrainment submodule and the phase control submodule. The mutual entrainment submodule controls the left upper-limb motor phases θ_*m,l*_ such that the phase difference Δθ_*m,l*_ between the left upper-limb arm-swing phase θ_*h, l*_ and the left upper-limb motor phase θ_*m,l*_ converges to 0°, where Δθ_*m,l*_ = θ_*h,l*_ – θ_*m,l*_. The phase θ_*h,l*_ is defined as the angle measured from the rearmost position of the left upper limb, with the rearmost position corresponding to 0°. The phase θ_*m,l*_ is defined as the motor phase that corresponds to the angle measured from the rearmost position. The submodule also controls the right upper-limb motor phase in the same manner, in terms of the variables θ_*m,r*_, Δθ_*m,r*_, and θ_*h, r*_.

The phase control submodule comprises two nonlinear coupled oscillators based on the Kuramoto model (Kuramoto, 1984), which can be represented as:

(1) $$\begin{array}{r} {\dot{\theta }}_{m,l}=\omega_{m,l}+K_{lr}\sin\left(\theta_{m,r}-\theta_{m,l}\right)+K_{m}\sin\left(\Delta \theta_{m,l}\right), \end{array}$$

(2) $$\begin{array}{r} {\dot{\theta }}_{m,r}=\omega_{m,r}+K_{lr}\sin\left(\theta_{m,l}-\theta_{m,r}\right)+K_{m}\sin\left(\Delta \theta_{m,r}\right). \end{array}$$

Here, ω_*m,l*_ and ω_*m,r*_are the variable intrinsic angular frequencies of the upper-limb motors. *K*_*lr*_is the coupling between the left and right upper-limb motor phases and *K*_*m*_denotes the strength of the phase difference convergence between the upper limbs and motors. In this study, we set *K*_*lr*_ = 5.0 and *K*_*m*_ = 0.5, following the parameter values used in the previous WalkMate framework (Miyake, 2009). Figure 2B is a schematic diagram showing the relationships between all phases and phase differences used in the phase control module.

The phase control submodule controls the intrinsic angular frequencies ω_*m,l*_ and ω_*m,r*_ of the upper-limb motors by converging the phase differences Δθ_*m,l*_ and Δθ_*m,r*_ to a target phase difference Δθ_*d*_. This operation of this submodule can be represented as:

(3) $$\begin{array}{r} {\dot{\omega }}_{m,l}=\varepsilon \text{sin}\left(\Delta \theta_{m,l}-\Delta \theta_{d}\right), \end{array}$$

(4) $$\begin{array}{r} {\dot{\omega }}_{m,r}=\varepsilon \text{sin}\left(\Delta \theta_{m,r}-\Delta \theta_{d}\right). \end{array}$$

Here, ε (>0) is a control gain. In this study, we initialize ε to 0.16, Δθ_*d*_ to 0°, and both ω_*m,l*_ and ω_*m,r*_ to 4.0 rad/s, because the frequency of complete human gait cycles is about 1.0 Hz.

### Subject Phase-input Module

This module receives the subject's upper-limb angular displacement from the upper-limb DC motor encoders. The control algorithm comprises four steps. First, both upper-limb motors are activated after four complete arm-swing cycles. At that time, both upper limbs are at their rearmost position (i.e., θ_*h, l*_ = 0° and θ_*h, r*_ = 0°). Second, the phase differences between each upper-limb phase and its corresponding upper-limb motor phase, (i.e., Δθ_*m,l*_ and Δθ_*m,r*_) are updated in real time such that the phase differences converge to the target phase difference Δθ_*d*_ = 0°. Third, the intrinsic angular frequencies of both upper-limb motors, ω_*m,l*_ and ω_*m,r*_, are updated using Equations (3) and (4) with the phase differences being updated from the second step. Fourth, the upper-limb motor phases θ_*m,l*_ and θ_*m,r*_ are updated using Equations (1) and (2) with the intrinsic angular frequencies updated from the third step. This cycle repeats from the second to the fourth steps during walking. Finally, the upper-limb motors stop when the operator switches off the exoskeleton using the Android phone controller or the end-user switches it off using the external switch. Figure 2C represents a flowchart of the software control algorithm for the wearable exoskeleton.

### Motor-Torque Output Module

**Figure 3:**
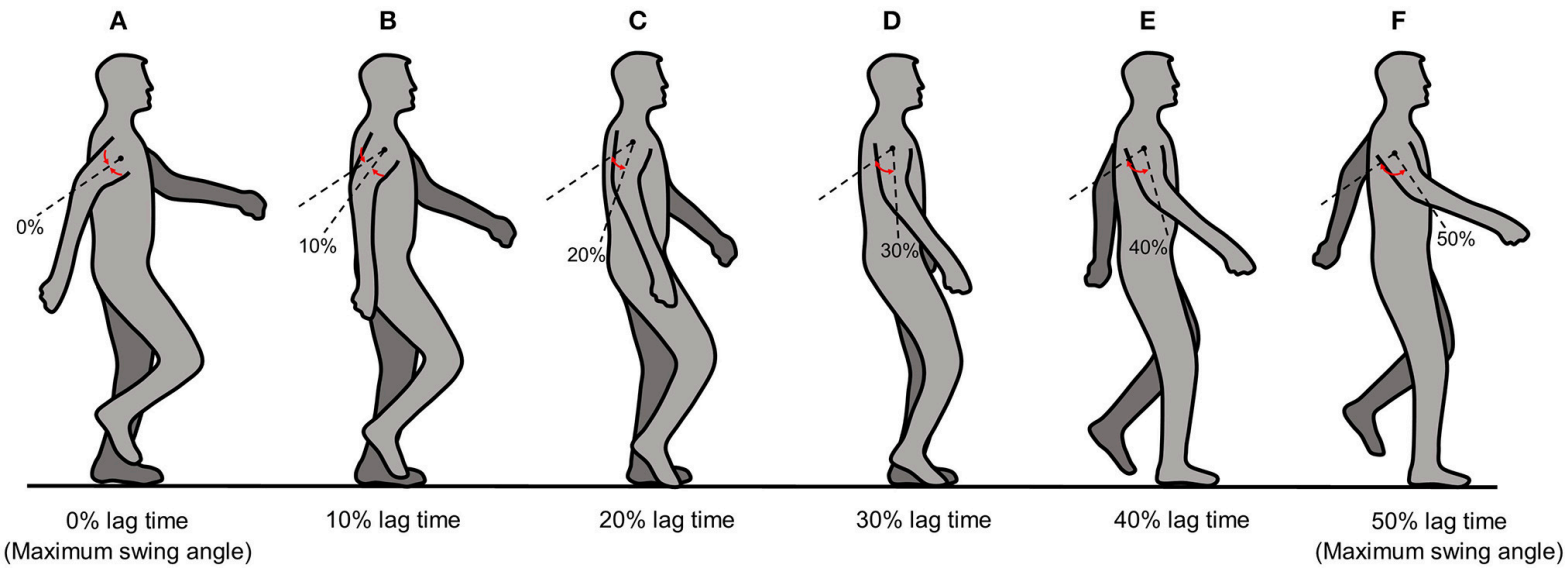
Schematic diagram of the upper-limb-assist conditions for various lag times: **(A)** 0% lag time, **(B)** 10% lag time, **(C)** 20% lag time, **(D)** 30% lag time, **(E)** 40% lag time, **(F)** 50% lag time.

This module follows each arm swing during one complete human gait cycle with temporal control of the motor torque. The upper-limb motor torque is applied at the contact point between the upper limb and the adjustable harness (i.e., just above the elbow joint). The phase of the output motor torque differs from the subject's arm-swing phase with respect to the rearmost position by an amount (i.e., lag time) that can be varied from 0 to 50% of one complete arm-swing cycle. The upper-limb-assist conditions for 0 and 50% lag times represent the output motor torque being applied to the upper limb at its rearmost and foremost positions, respectively. However, because the foremost position corresponds to the exact time that the arm reverses its swing direction, using a 50% lag time might cause instability in the subject. We therefore restricted the maximum lag time to 40% in this study, where the upper limb is at an arm-swing position between the subject's frontal plane and the limb's foremost position. Figures 3A–F show schematically the upper-limb-assist conditions for various lag times.

## Experiment

### Ethics Statement

The experiment protocol in this study was approved by the Ethics Committee at the Tokyo Institute of Technology through written consent. We recruited healthy elderly subjects from the Machida Silver Center in Tokyo, Japan. They were all free from documented neurological disorders. Before the start of the experiment, all participants were briefed about the experimental procedures and written informed consent was obtained.

### Participants

We evaluated the wearable exoskeleton by conducting walking experiments with 12 healthy elderly male subjects. The mean age of the subjects was 74.5 ± 2.6 years. Their mean height and weight were 166.2 ± 5.1 cm and 63.3 ± 10.1 kg, respectively (Data Sheet 2).

### Experimental Procedure

The experimental procedure comprised two steps. First, the subjects walked a horizontal distance of 55.4 m along a corridor at their own natural speed with a natural arm swing. This process established a baseline called the “free condition” (Data Sheet 2). Three complete trials were conducted under this condition. Second, three experimental sessions were conducted. In each experimental session, the subjects put on the wearable exoskeleton and walked at their own natural walking speed under upper-limb-assist conditions involving five different lag times (0, 10, 20, 30, and 40%) conducted randomly. For each condition, the subjects walked the same horizontal distance of 55.4 m along the corridor. Three complete trials were conducted for each condition. There was no rest time given between the first and second experimental sessions, but a rest time of about 5 min was given between the second and third sessions. The experiment ended when the subjects had completed all three experimental sessions.

During the experiment, each subject also wore a wearable sensor (TSND121; ATR-Promotion, Japan) on each of his lower limbs. The sensor comprised an accelerometer and a gyroscope capable of measuring acceleration and angular velocity in all three dimensions. The sensor was rigidly secured to the lower limb using elastic Velcro belts at a vertical height of 15.0 cm above the knee joint on the sagittal plane. The time-series data from the wearable sensors were recorded during the experiment using a sampling frequency of 100 Hz and then transmitted to a portable laptop computer (Dell Latitude, E5440; Dell, USA) via Bluetooth for off-line analysis. The sampling precision of the angular velocity is 0.01 degree per second. The time taken for each subject to complete each experimental trial was also measured using a digital stopwatch (HS44-001; Citizen, Japan) to calculate the average walking speed of each subject.

### Gait Analysis

We performed a gait analysis of the left and right hip-swing angular displacements and hip-swing periods using the time-series data obtained from the wearable sensors on the left and right lower limbs to evaluate the effect of the interactive rhythmic stimulation applied to the upper limbs under all conditions. The gait analysis comprised eight steps (Data Sheet 1).

First, we applied a fourth-order zero-phase shift Butterworth low-pass filter with a cut-off frequency of 6.0 Hz to the time-series data for the hip-swing angular velocity in the sagittal plane, because it has been reported that the frequencies for normal gait are within a narrow band, with the upper limit between 4.0–6.0 Hz (Winter et al., 1974; Angeloni et al., 1994). Second, we performed a simple numerical integration of the time-series data on the hip-swing angular velocities using the trapezoidal rule to obtain the left and right hip-swing angular displacements θ_*z, l*_(*t*) and θ_*z, r*_(*t*). Third, we applied a small recursive filter to θ_*z, l*_(*t*) and θ_*z, r*_(*t*) to correct for the accumulation drift error caused by the numerical integration in the second step. Fourth, we extracted each peak and trough of the corrected time-series data for the hip-swing angular displacements using a peak-detection algorithm to calculate the peak-to-peak hip-swing amplitudes for all complete stable gait cycles in each complete trial. Fifth, we calculated the mean peak-to-peak amplitude of the time-series data for the left and right hip-swing angular displacements, removing the first and last five gait cycles because of acceleration and deceleration issues, to obtain the mean left and right hip-swing amplitudes. Sixth, we calculated the mean hip-swing amplitude as the mean value of the mean left and right hip-swing amplitudes. Seventh, we calculated the time difference between two consecutive peaks and troughs of the time-series data for the left and right hip-swing angular displacements to calculate the mean left and right hip-swing periods. Finally, we calculated the mean hip-swing period, which is the mean of the mean left and right hip-swing periods. Postprocessing of the time-series data from the wearable sensors was performed using the Scientific Computing Library, SciPy in Python (version 2.7) and numerical analysis of the time-series data was performed using a Microsoft Excel spreadsheet.

### Statistical Analysis

Statistical analysis comprised a two-step process. First, we performed a statistical analysis of the mean hip-swing angular displacement and the mean hip-swing period between the free condition and each of the upper-limb-assist conditions, using the Friedman test because of the nonparametric distribution of the numerical data (*n* = 12). Second, if a statistically significant difference existed from the previous test, we performed a multiple pairwise Wilcoxon signed-rank test. To address the type-I and type-II errors introduced as a result of multiple pairwise testing, we performed a correction to the statistical test using the Bonferroni correction (Bland and Altman, 1995). All statistical analyses were performed using R (version 3.3.3). A statistically significant difference between the free condition and each of the upper-limb-assist conditions was confirmed at *p* < 0.05.

## Results

**Figure 4:**
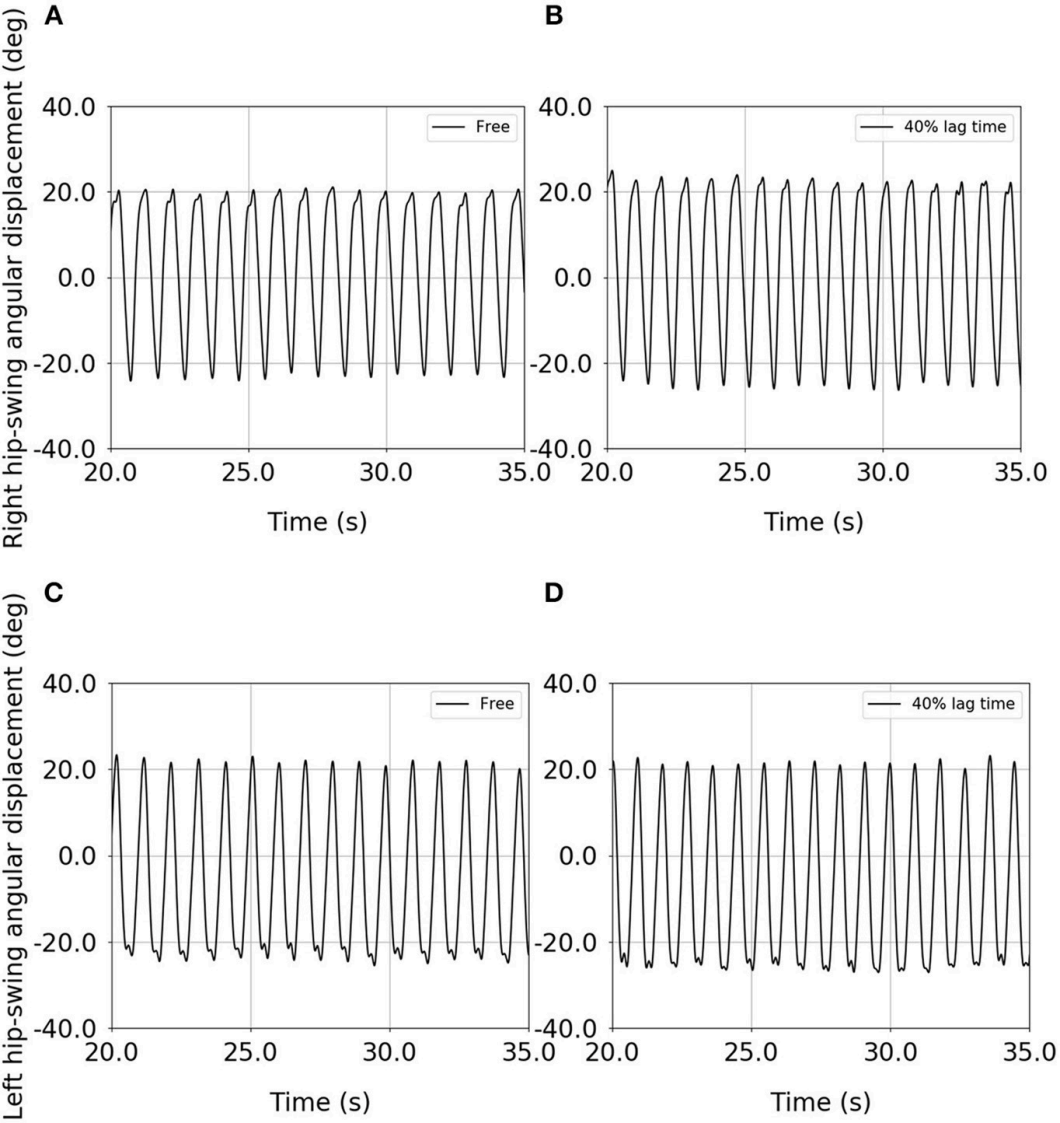
Example of the time-series data for the right and left hip-swing angular displacements for an elderly subject: **(A)** right hip-swing angular displacement under the free condition, **(B)** right hip-swing angular displacement under the upper-limb-assist condition with a 40% lag time, **(C)** left hip-swing angular displacement under the free condition, **(D)** left hip-swing angular displacement under the upper-limb-assist condition with a 40% lag time.

Figures 4A–D shows examples of the time-series data for both the right and left hip-swing angular displacements, for both the free condition and the upper-limb-assist condition with a 40% lag time, for one elderly subject (Data Sheet 2). These results show that the peak-to-peak amplitude for each of the right and left hip-swing angular displacements is higher for the upper-limb-assist condition with a 40% lag time than for the free condition.

**Figure 5:**
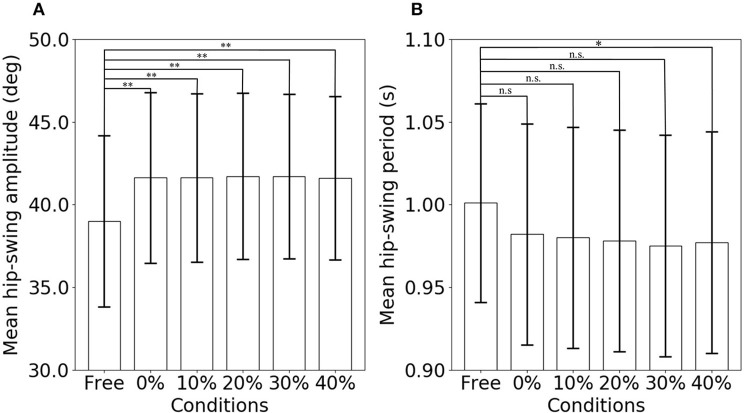
Statistical analysis of the mean hip-swing amplitude and the mean hip-swing period for the free condition and for each of the upper-limb-assist conditions with elderly subjects: **(A)** mean hip-swing amplitude, **(B)** mean hip-swing period (*p < 0.05; **p < 0.01).

Figure 5A shows the results of the statistical analysis of the mean hip-swing amplitude for the elderly subjects (Data Sheet 2). Table 1 gives the mean hip-swing amplitude and the mean hip-swing period for each experimental condition, and the *p*-value for the upper-limb-assist conditions with respect to the free condition. A Friedman test revealed a significant effect of upper-limb conditions on the hip-swing amplitude [*A*^2^(5) = 27.1, *p* < 0.01]. A *post-hoc* test using Wilcoxon signed-rank test with Bonferroni correction showed a statistically significant difference between the free condition and each of the upper-limb-assist conditions with respect to the mean hip-swing amplitude under all conditions (*p* = 0.0024). In particular, the mean hip-swing amplitude shows a statistically significant increase of about 2.8° from the free condition to each of the upper-limb-assist conditions. The results show an increase in the mean hip-swing amplitude for each of the upper-limb-assist conditions against the free condition.

**Table 1:** Mean and *p*-values for the mean hip-swing amplitude and the mean hip-swing period for each experimental condition.

**Parameter**	**Condition**	**Mean (±SD)**	***p*-value**
Hip-swing amplitude (°)	Free	38.90 (± 5.24)	–
	0% lag time	41.62 (± 5.16)	0.0024^**^
	10% lag time	41.63 (± 5.10)	0.0024^**^
	20% lag time	41.72 (± 5.04)	0.0024^**^
	30% lag time	41.69 (± 4.98)	0.0024^**^
	40% lag time	41.60 (± 4.94)	0.0024^**^
Hip-swing period (ms)	Free	1000.5 (± 60.4)	–
	0% lag time	982.3 (± 67.5)	0.088
	10% lag time	980.1 (± 66.8)	0.081
	20% lag time	978.0 (± 66.7)	0.061
	30% lag time	974.9 (± 67.3)	0.061
	40% lag time	977.1 (± 67.3)	0.024^*^

* p < 0.05;

** *p < 0.01*.

Figure 5B shows the results of the statistical analysis of the mean hip-swing period for the elderly subjects (File S5). A Friedman test revealed a significant effect of upper-limb conditions on the hip-swing period (*A*^2^(5) = 17.2, *p* = 0.00412). A *post hoc* test using Wilcoxon signed-rank test with Bonferroni correction showed a statistically significant difference between the free condition and the upper-limb-assist condition with a 40% lag time (*p* = 0.024), where the mean hip-swing period decreased on average by 23 ms. However, no statistically significant differences were observed for other upper-limb-assist conditions (*p* = 0.088, 0.081, 0.061, and 0.061, respectively).

## Discussion

The gait-assist wearable exoskeleton developed in this study, the WalkMate, applied interactive rhythmic stimulation to the elderly subjects' upper limbs, aiming to support their gait based on the principle of mutual entrainment (i.e., interpersonal coordination) in human–robot interaction and upper–lower–limbs' neural coupling (i.e., intrapersonal coordination) as mediated by central pattern generators. We postulate that this approach would provide support to the lower limbs based on the intrinsic mechanisms of upper–lower–limbs' coordination in human locomotion. This support is in contrast with that of previous power-assist wearable exoskeletons that aim to provide direct torque to the lower limbs. To verify our hypothesis, we investigated the effect of such stimulation on spatial (i.e., hip-swing amplitude) and temporal (i.e., hip-swing period) gait parameters by conducting walking experiments with healthy elderly subjects.

It has been reported that changes in gait pattern with increasing age are associated with decreasing muscle strength and that there is a need for increased stability during locomotion with increasing age (Nigg et al., 1994). In addition, it has been reported that age causes a redistribution of joint torques and powers, with the elderly using their hip extensors more than young adults walking at the same speed (DeVita and Hortobagyi, 2000). We feel that it is important to assist healthy elderly subjects to regain their normal gait ability as a result of aging to assist them in their activities of daily living. In this regard, we consider our gait-assist wearable exoskeleton able to meet this objective and encourage voluntary efforts (i.e., active mode) by the subjects, thereby providing appropriate support to the lower limbs.

Although many power-assist wearable exoskeletons have been developed, these exoskeletons aim to provide a direct torque to the wearer's lower limb by controlling the gait trajectory to reach a target trajectory (Kawamoto et al., 2003; Riener et al., 2005; Veneman et al., 2007; Kim et al., 2010; Barbareschi et al., 2015; Bortole et al., 2015; Chen et al., 2017; Deng et al., 2017; Lerner et al., 2017; Choi et al., 2018). Moreover, there is no direct comparison between the baseline condition (i.e., free walking) and the robot-assist condition. Hence, the effectiveness of the direct power-assist support of these wearable exoskeletons to the lower limbs remains unclear.

In contrast, in this study, we conducted walking experiments with healthy elderly subjects using the baseline condition (i.e., free walking) and upper-limb-assist conditions at different lag times. The results showed a statistically significant increase in the mean hip-swing angular displacement for all upper-limb-assist conditions compared with the free condition, with a mean increment of about 7% (i.e., 2.8°). It has been reported that the step length was 4% shorter in healthy elderly adults compared with healthy young adults (DeVita and Hortobagyi, 2000). In addition, it has been reported that the stride length was 6.7% (Ostrosky et al., 1994) and 10% (Winter et al., 1990) shorter in healthy elderly adults compared with healthy young adults, respectively. In this study, we observed a statistically significant increase in the hip-swing amplitude by about 7% between the free condition and each upper-limb-assist condition at different lag times with the healthy elderly subjects. Hence, the result indicates that interactive rhythmic stimulation to the upper limbs could effectively increase the hip-swing amplitude for elderly adults' gait to be comparable to the gait of healthy young adults.

In addition, Lerner et al. (2017) reported that power-assist support to the wearer's knee joint showed a statistically significant increase in the peak hip extension of children with cerebral palsy by 8° between the baseline walking condition (i.e., free walking) and the power-assist condition. Although the increase in the mean hip-swing amplitude in our present study is smaller than that measured in the previous study (Lerner et al., 2017), it should be noted that all the participants in the previous study are unhealthy subjects, whereas all our participants are healthy subjects. Hence, the result supports the hypothesis that applying appropriate interactive rhythmic stimulation to the elderly adult's upper limbs would affect their lower limbs' gait patterns as manifested through an increase in the hip-swing amplitude. We can speculate from the results that the mutual entrainment resulting from such stimulation could activate the central pattern generators of the upper limbs, thereby stimulating the lower limbs as mediated through a cooperative relationship (intra-synchronization) between the upper and lower limbs. Such a relationship has been reported in terms of upper–1ower–imbs' neural coupling in many previous studies (Delwaide et al., 1977; Baldissera et al., 1982, 1988; Guadagnoli et al., 2000; Dietz et al., 2001; Zehr et al., 2001; Zehr and Haridas, 2003; Frigon et al., 2004; Huang and Ferris, 2004, 2009; Zehr and Duysens, 2004; Kawashima et al., 2008; Strausser and Kazerooni, 2011). In particular, the soleus muscles of the lower limbs could be activated (Huang and Ferris, 2004, 2009; Kawashima et al., 2008) and the presynaptic inhibition could be suppressed (Zehr and Duysens, 2004) by the stimulation of the upper limbs via the upper–lower–limbs' neural coupling.

In a second main result, we observed a statistically significant decrease in the mean hip-swing period of the subjects for the upper-limb-assist condition with a 40% lag time compared with the free condition. However, there is no statistically significant difference between the upper-limb-assist condition with a 0, 10, 20, and 30% lag time, and the free condition. It has been reported that the stride velocity of healthy elderly adults is 7.9% lower than that of healthy young adults (Ostrosky et al., 1994). In addition, it has been reported that there are 7.7% (DeVita and Hortobagyi, 2000) and 2.3% (Krasovsky et al., 2014) reported decreases in the swing time of healthy elderly adults compared with healthy young adults. In this study, we observed a statistically significant decrease in the hip-swing period by 2.3% (i.e., 23 ms) between the free condition compared with the upper-limb-assist condition at a 40% lag time. Although the decrease in the hip-swing period (i.e., increase in the gait speed) is smaller in our present study compared with that measured by DeVita and Hortobagyi (2000), it is comparable with the results presented by Krasovsky et al. (2014). Hence, the result indicates that interactive rhythmic stimulation to the upper limbs could increase the gait speed of the elderly to be comparable to the gait of healthy young adults.

Although this decrease in the hip-swing period is small, the result indicates that interactive rhythmic stimulation to the upper limbs at an optimal arm-swing position can increase the arm-swing activity. Because it has been reported in previous studies that an increase in arm-swing activity increases the gait speed (Eke-Okoro et al., 1997; Marks, 1997; Long et al., 2011), the results indicate the possibility that interactive rhythmic stimulation to the upper limbs could increase the gait speed with these healthy elderly subjects due to an increase in arm-swing activity, particularly at a 40% lag time. Moreover, it has been reported in a previous study that postural changes of the upper limb affect the reflex transmission of the lower limbs, which is maximal when the upper limb is at an angle of 45° with respect to the frontal plane (Delwaide et al., 1977). This position corresponds to the arm-swing position of the upper limbs at a 40% lag time in our study. It would be possible that stimulation at this arm-swing position could activate the soleus reflex of the lower limbs significantly, again leading to increased gait speed. Therefore, the effect of the stimulation on the gait speed might be optimized for the 40% lag time.

However, there are some limitations in our present study. First, the weight of the gait-assist wearable exoskeleton used is heavy (5.8 kg) for the elderly subjects. Although our results showed a statistically significant increase in the hip-swing amplitude and gait speed of these subjects, the marginal change indicates that the weight of the wearable exoskeleton might have counteracted the benefits of the interactive rhythmic stimulation. Because of this possibility, the development of a lightweight gait-assist wearable exoskeleton for the elderly is underway. Second, the sample size of our elderly subjects (*n* = 12) can be considered small. Our preliminary results showed that some inter-subject variability exists even for healthy elderly subjects in the hip-swing amplitude and hip-swing period. Thus, there is a need to increase the sample size of the elderly subjects to minimize the effect of this variability. Third, we have only conducted three experimental sessions for each experimental condition on the same day. In order to account for the effect of neuroplasticity, there is a need to conduct more experimental sessions over a longer time frame including “free” walking experiments immediately after walking experiments using the wearable exoskeleton (i.e., “wash-out” effect).

Despite the aforementioned limitations, our results suggest that the WalkMate gait-assist wearable exoskeleton succeeds in generating interactive rhythmic stimulation in synchronization with an elderly subject's arm swing, based on the mutual entrainment principle. Moreover, this principle plays an important role in establishing intrapersonal coordination between the upper and lower limbs of the subjects. In addition, it should also be noted that the autonomous gait ability of subjects has been an important aspect of the establishment of this principle.

We have three research plans for the future. First, we recruited male-only subjects for this study. Because there are gender differences in gait patterns because of a different range of bodily mass and the position of the center of gravity, we will evaluate the effect of stimulation to the upper limbs on female subjects' gait. Second, we plan to conduct experiments on walking for longer periods to determine the “wash-out” effects of the upper-limb stimulation. Finally, it has been reported that elderly adults with lower extremity dysfunction rely excessively on the passive action of the hip flexors to provide propulsion in the late stance to enhance stability (McGibbon and Krebs, 2004). Hence, we aim to extend our experiments to this group of elderly adults as well as patients with neurological disorders such as hemiplegia and Parkinson's disease.

## Ethics Statement

The experiment protocol in this study was approved by the Ethics Committee at the Tokyo Institute of Technology through written consent. We recruited healthy elderly subjects from the Machida Silver Center in Tokyo, Japan. They were all free from documented neurological disorders. Before the start of the experiment, all participants were briefed about the experimental procedures and written informed consent was obtained.

## Author Contributions

RY, TN, KO, and YM: conceptualization; RY: data curation; RY: formal analysis; YM: funding acquisition; RY: investigation; RY, TN, KO, and YM: methodology; YM: project administration; YH, MS, MN, and KI: resource; YH, MS, and MN: software; KO and YM: supervision; RY: validation, visualization, and writing (original draft preparation); RY, KO, and YM: writing (review and editing).

### Conflict of Interest Statement

MS, MN, and KI were employed by company Kikuchi Seisakusho Co. Ltd. The remaining authors declare that the research was conducted in the absence of any commercial or financial relationships that could be construed as a potential conflict of interest.

## References

[B1] Angeloni C. Riley P. O. Krebs D. E. (1994). Frequency content of whole body gait kinematic data. IEEE Trans. Rehabil. Eng. 1994, 40–46. 10.1109/86.296343

[B2] Baldissera F. Cavallari P. Civaschi P. (1982). Preferential coupling between voluntary movements of ipsilateral limbs. Neurosci. Lett. 34, 95–100. 10.1016/0304-3940(82)90098-2 7162702

[B3] Baldissera F. Cavallari P. Leocani L. (1988). Cyclic modulation of the H-reflex in a wrist flexor during rhythmic flexion-extension movements of the ipsilateral foot. Exp. Brain Res. 118, 427–430. 10.1007/s002210050297 9497150

[B4] Barbareschi G. Richard R. Thornton M. Carlson T. (2015). Statically vs. dynamically balanced gait: analysis of a robotic exoskeleton compared with a human, in Conference Procceedings IEEE Engineering Medical Biology Socity (Milan), 6728–6731. 10.1109/EMBC.2015.7319937 26737837

[B5] Bland J. M. Altman D. G. (1995). Multiple significance tests: the bonferroni method. BMJ 310:170. 10.1136/bmj.310.6973.170 7833759 PMC2548561

[B6] Bortole M. Venkatakrishnan A. Zhu F. Moreno J. C. Francisco G. E. Pons J. L.. (2015). The H2 robotic exoskeleton for gait rehabilitation after stroke: Early findings from a clinical study. J. NeuroEng. Rehabil. 12:54. 10.1186/s12984-015-0048-y 26076696 PMC4469252

[B7] Chen B. Zhong C. H. Zhao X. Ma H. Guan X. Li X. (2017). A wearable exoskeleton suit for motion assistance to paralyzed patients. J. Ortho. Tran. 11, 7–18. 10.1016/j.jot.2017.02.007 PMC5866401 29662765

[B8] Choi H. Seo K. Hyung S. Shim Y. Lim S. (2018). Compact hip-force sensor for a gait-assistance exoskeleton system. Sensors 18:566. 10.3390/s18020566 29438300 PMC5856075

[B9] Cohen A. H. Holmes P. J. Rand R. H. (1982). The nature of the coupling between segmental oscillators of the lamprey spinal generator for locomotion: a mathematical model. J. Math. Biol. 13, 345–369. 10.1007/BF00276069 7057117

[B10] Delwaide P. J. Figiel C. Richelle C. (1977). Effects of postural changes of the upper limb on reflex transmission in the lower limb. Cervicolumbar reflex interactions in man. J. Neurol. Neurosurg. Psychiatry 40, 616–621. 10.1136/jnnp.40.6.616 903777 PMC492771

[B11] Deng J. Wang P. Li M. Guo W. Zha F. Wang X. (2017). Structure design of active power-assist lower limb exoskeleton APAL robot. Adv. Mech. Eng. 9, 1–11. 10.1177/1687814017735791

[B12] DeVita P. Hortobagyi T. (2000). Age causes a redistribution of joint torques and powers during gait. J. Appl. Physiol. 88, 1804–1811. 10.1152/jappl.2000.88.5.1804 10797145

[B13] Dietz V. Fouad K. Bastiaanse C. M. (2001). Neuronal coordination of arm and leg movements during human locomotion. Eur. J. Neurosci. 14, 1906–1914. 10.1046/j.0953-816x.2001.01813.x 11860485

[B14] Eke-Okoro S. T. Gregoric M. Larsson L. E. (1997). Alterations in gait resulting from deliberate changes of arm-swing amplitude and phase. Clin. Biomech. 12, 516–521. 10.1016/S0268-0033(97)00050-8 11415762

[B15] Frigon A. Collins D. F. Zehr E. P. (2004). Effect of rhythmic arm movement on reflexes in the legs: modulation of soleus H-reflexes and somatosensory conditioning. J. Neurophysiol. 91, 1516–1523. 10.1152/jn.00695.2003 14657191

[B16] Guadagnoli M. A. Etnyre B. Rodrigue M. L. (2000). A test of a dual central pattern generator hypothesis for subcortical control of locomotion. J. Electromyogr. Kinesiol. 10, 241–247. 10.1016/S1050-6411(00)00018-3 10969197

[B17] Huang H. J. Ferris D. P. (2009). Upper and lower limb muscle activation is bidirectionally and ipsilaterally coupled. Med. Sci. Sports Exerc. 41, 1778–1789. 10.1249/MSS.0b013e31819f75a7 19657291 PMC2769567

[B18] Huang J. H. Ferris D. P. (2004). Neural coupling between upper and lower limbs during recumbent stepping. J. Appl. Physiol. 97, 1299–2004. 10.1152/japplphysiol.01350.2003 15180979

[B19] Kawamoto H. Lee S. Kanbe S. Sankai Y. (2003). Power assist method for HAL-3 using EMG-based feedback controller, in Conference Procceeding IEEE International Conference System Man and Cybernetics (San Francisco, CA), 1648–1653. 10.1109/ICSMC.2003.1244649

[B20] Kawashima N. Nozaki D. Abe M. O. Nakazawa K. (2008). Shaping appropriate locomotive motor output through interlimb neural pathway within spinal cord in humans. J. Neurophysiol. 99, 2946–2955. 10.1152/jn.00020.2008 18450579

[B21] Kim S. H. Banala S. K. Brackbill E. A. Agrawal S. K. Krishnamoorthy V. Scholz J. P. (2010). Robot-assist modifications of gait in healthy individuals. Exp. Brain Res. 202, 809–824. 10.1007/s00221-010-2187-5 20186402

[B22] Krasovsky T. Lamontagne A. Feldman A. G. Levin M. G. (2014). Effects of walking speed on gait stability and interlimb coordination in younger and older adults. Gait Posture 39, 378–385. 10.1016/j.gaitpost.2013.08.011 24008010

[B23] Kuramoto Y. (1984). Chemical Oscillations, Waves and Turbulence (Heidelberg: Springer-Verlag).

[B24] Lerner Z. F. Damiano D. L. Bulea T. C. (2017). The effects of exoskeleton assisted knee extension on lower-extremity gait kinematics, kinetics, and muscle activity in children with cerebral palsy. Sci. Rep. 7:13512. 10.1038/s41598-017-13554-2 29044202 PMC5647342

[B25] Long J. T. Groner J. B. Eastwood D. C. Dillingham T. R. Grover P. Harris G. F. (2011). Implications of arm restraint on lower extremity kinetics during gait. J. Exp. Clin. Med. 3, 200–206. 10.1016/j.jecm.2011.09.006

[B26] Marks R. (1997). The effects of restricted arm swing during normal locomotion. Biomed. Sci. Instrum. 33, 209–215. 9731361

[B27] McGibbon C. A. Krebs D. E. (2004). Discriminating age and disability effects in locomotion: neuromuscular adaptations in musculoskeletal pathology. J. Appl. Physiol. 96, 149–160. 10.1152/japplphysiol.00422.2003 12949019

[B28] Miyake Y. (2009). Interpersonal synchronization of body motion and the Walk-Mate walking support robot. IEEE Trans. Robot 25, 638–644. 10.1109/TRO.2009.2020350

[B29] Nigg B. M. Fisher V. Ronsky J. L. (1994). Gait characteristics as a function of age and gender. Gait Posture 2, 213–220. 10.1016/0966-6362(94)90106-6

[B30] Ostrosky K. M. VanSwearingen J. M. Burdett R. G. Gee Z. (1994). A comparison of gait characteristics in young and old subjects. Phys. Ther. 74, 637–644. 10.1093/ptj/74.7.637 8016196

[B31] Riener R. Lunenburger L. Jezernik S. Anderschitz M. Colombo G. Dietz V. (2005). Patient-cooperative strategies for robot-aided treadmill training: First experimental results. IEEE Trans. Neural. Syst. Rehabil. Eng. 13, 380–394. 10.1109/TNSRE.2005.848628 16200761

[B32] Strausser K. A. Kazerooni H. (2011). The development and testing of a human machine interface for a mobile medical exoskeleton, in Conference Proceeding IEEE/RSJ International Conference Intelligent Robots and System (San Francisco, CA), 4911–4916. 10.1109/IROS.2011.6095025

[B33] Taga G. Yamaguchi Y. Shimizu H. (1991). Self-organized control of bipedal locomotion by neutral oscillators in unpredictable environment. Biol. Cybern. 65, 147–159. 10.1007/BF00198086 1912008

[B34] Veneman J. F. Kruidhof R. Hekman E. E. G. Ekkelenkamp R. Van Asseldonk E. H. F. Van der Kooji H. (2007). Design and evaluation of the LOPES exoskeleton robot for interactive gait rehabilitation. IEEE Trans. Neural. Syst. Rehabil. Eng. 15, 379–386. 10.1109/TNSRE.2007.903919 17894270

[B35] Winter D. A. Patia A. E. Frank J. S. Walt S. E. (1990). Biomechanical walking pattern changes in the fit and healthy elderly. Phys. Ther. 70, 340–347. 10.1093/ptj/70.6.340 2345777

[B36] Winter D. A. Sidwall H. G. Hobson D. A. (1974). Measurement and reduction of noise in kinematics of locomotion. J. Biomech. 7, 157–159. 10.1016/0021-9290(74)90056-6 4837552

[B37] Yuasa H. Ito M. (1990). Coordination of many oscillators and generation of locomotory patterns. Biol. Cybern. 63, 177–184. 10.1007/BF00195856

[B38] Zehr E. P. Collins D. F. Chua R. (2001). Human interlimb reflexes evoked by electrical stimulation of cutaneous nerves innervating the hand and foot. Exp. Brain Res. 140, 495–504. 10.1007/s002210100857 11685403

[B39] Zehr E. P. Duysens J. (2004). Regulation of arm and leg movement during human locomotion. Neuroscientist 10, 347–361. 10.1177/1073858404264680 15271262

[B40] Zehr E. P. Haridas C. (2003). Modulation of cutaneous reflexes in arm muscles during walking: further evidence of similar control mechanisms for rhythmic human arm and leg movements. Exp. Brain Res. 149, 260–266. 10.1007/s00221-003-1377-9 12610695

