# Supplementary material for: Gait-Assist Wearable Robot Using Interactive Rhythmic Stimulation to the Upper Limbs
Source: Front Robot AI. 2019 Apr 24;6:25. doi: 10.3389/frobt.2019.00025 (PMC7805921; doi:10.3389/frobt.2019.00025)
Supplement: Supplementary file 1 [file Data_Sheet_1.PDF]

# Supplementary information for gait analysis

A simple numerical integration of the hip-swing angular velocity time-series data in the sagittal plane using trapezoidal rule to obtain the hip-swing angular displacement can be represented as:

$$\theta_z = \int_0^t \omega_z dt \qquad (1)$$

Here, $\theta_z$ is the hip-swing angular displacement in the sagittal plane, $t$ is the time interval for one complete hip-swing cycle, and $\omega_z$ is the hip-swing angular velocity in the sagittal plane.

A small recursive filter to $\theta_z$ to correct for accumulation drift error due to numerical integration can be represented as:

$$\theta_z^{'}(n) = \theta_z(n) - \theta_z(n-1) + K\theta_z(n-1) \qquad (2)$$

Here, $\theta_z^{'}(n)$ is the corrected hip-swing angular displacement in the sagittal plane, $n$ is the length of the hip-swing time-series data and $K$ is 0.995.

The mean left or right hip-swing amplitude was calculated by taking the mean of the peak-to-peak amplitude of the left or right hip-swing angular displacement time-series data, which can be represented as:

$$\overline{\theta}_h = \frac{1}{l}\sum_{n=i}^{l}[\theta_p(n) - \theta_t(n)] \qquad (3)$$

Here, $\overline{\theta}_h$ is the mean left or right hip-swing amplitude, $\theta_p$ and $\theta_t$ is the left or right hip-swing angular displacement at each peak and trough, respectively, and $l$ is the number of complete gait cycles excluding the first and last five complete gait cycles.

The mean left or right hip-swing period was calculated by taking the mean of the time difference between two consecutive peaks and troughs of the left or right hip-swing angular displacement time-series data, which can be represented as:

$$\overline{T_h} = \frac{1}{2l}\sum_{n=1}^{l}[(t_p(n+1) - t_p(n)) + (t_t(n+1) - t_t(n))] \quad (4)$$

Here, $\overline{T_h}$ is the mean left or right hip-swing period, $t_p$ and $t_t$ is the corresponding time of each peak and trough of the left or right hip-swing angular displacement, and $l$ is the number of complete gait cycles excluding the first and last five complete gait cycles.
