# Supplementary material for: Gait-Assist Wearable Robot Using Interactive Rhythmic Stimulation to the Upper Limbs
Source: Front Robot AI. 2019 Apr 24;6:25. doi: 10.3389/frobt.2019.00025 (PMC7805921; doi:10.3389/frobt.2019.00025)
Supplement: Supplementary file 2 [file Data_Sheet_2.PDF]

Supplementary Material

Gait-assist Wearable Robot Using Interactive Rhythmic Stimulation to the Upper Limbs

Robin Miao Sin Yap[1*], Ken-ichiro Ogawa[2], Yuki Hirobe[1], Terumasa Nagashima[1], Masatoshi Seki[3], Masayuki Nakayama[3], Ken Ichiryu[3], Yoshihiro Miyake[2]

*** Correspondence:**
Corresponding Author
robinyms78@gmail.com

1 Files

File S1 Age (years), height (cm), and weight (kg) of participants. The values represent mean ± SD.

File S2 Numerical data for the mean gait speeds of participants. “Free”, “0%”, “10%”, “20%”, “30%”, and “40%” refer to the average speed for the free condition and the five upper-limb-assist conditions with the stated lag times, respectively, in m/s.

File S3 Information details about the gait analysis. This file includes the mathematical equations used for the analysis of the hip-swing angular displacement and hip-swing period.

File S4 Numerical data for Figures 4(A) to 4(D). “Free” and “40%” refer to the right hip-swing angular displacement for the free condition and for the upper-limb-assist condition with a 40% lag time, respectively.

File S5 Numerical data for Figures 5(A) and 5(B). “Free”, “0%”, “10%”, “20%”, “30%”, and “40%” refer to the free condition and the five upper-limb-assist conditions with a 0%, 10%, 20%, 30%, and 40% lag time, respectively, in degrees. Figure 5(A) shows the mean hip-swing angular displacements. Figure 5(B) shows the mean hip-swing periods. The values represent mean ± SD.
