# Supplementary figures and images for: Gait-Assist Wearable Robot Using Interactive Rhythmic Stimulation to the Upper Limbs
Source: Front Robot AI. 2019 Apr 24;6:25. doi: 10.3389/frobt.2019.00025 (PMC7805921; doi:10.3389/frobt.2019.00025)

**A**

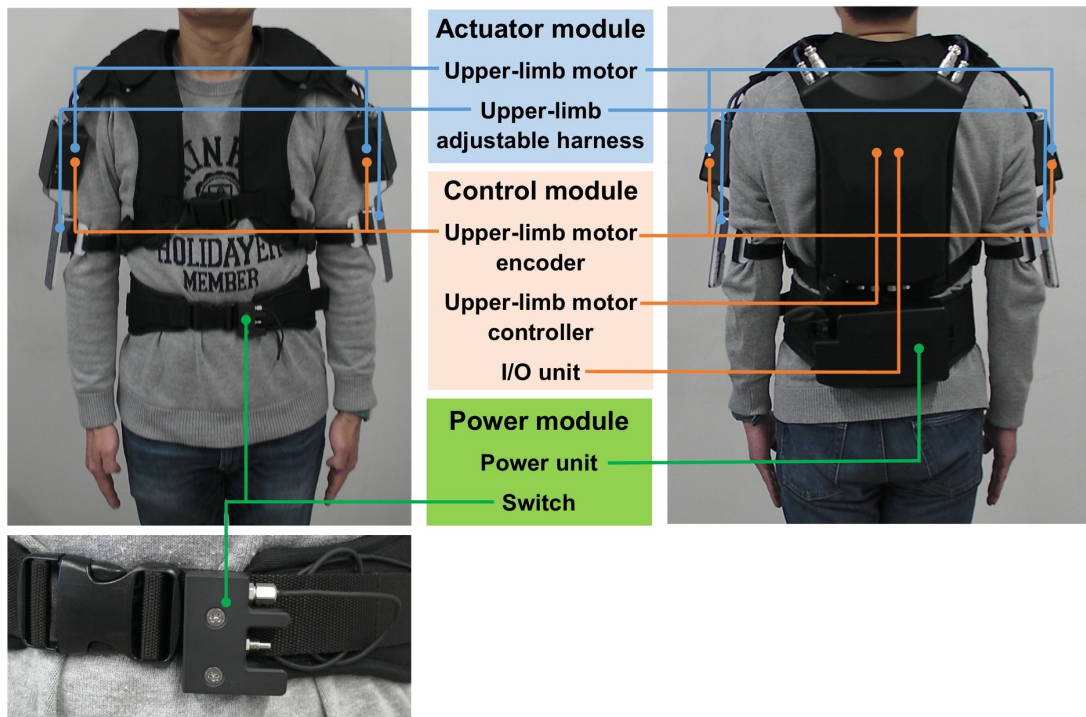

**B**

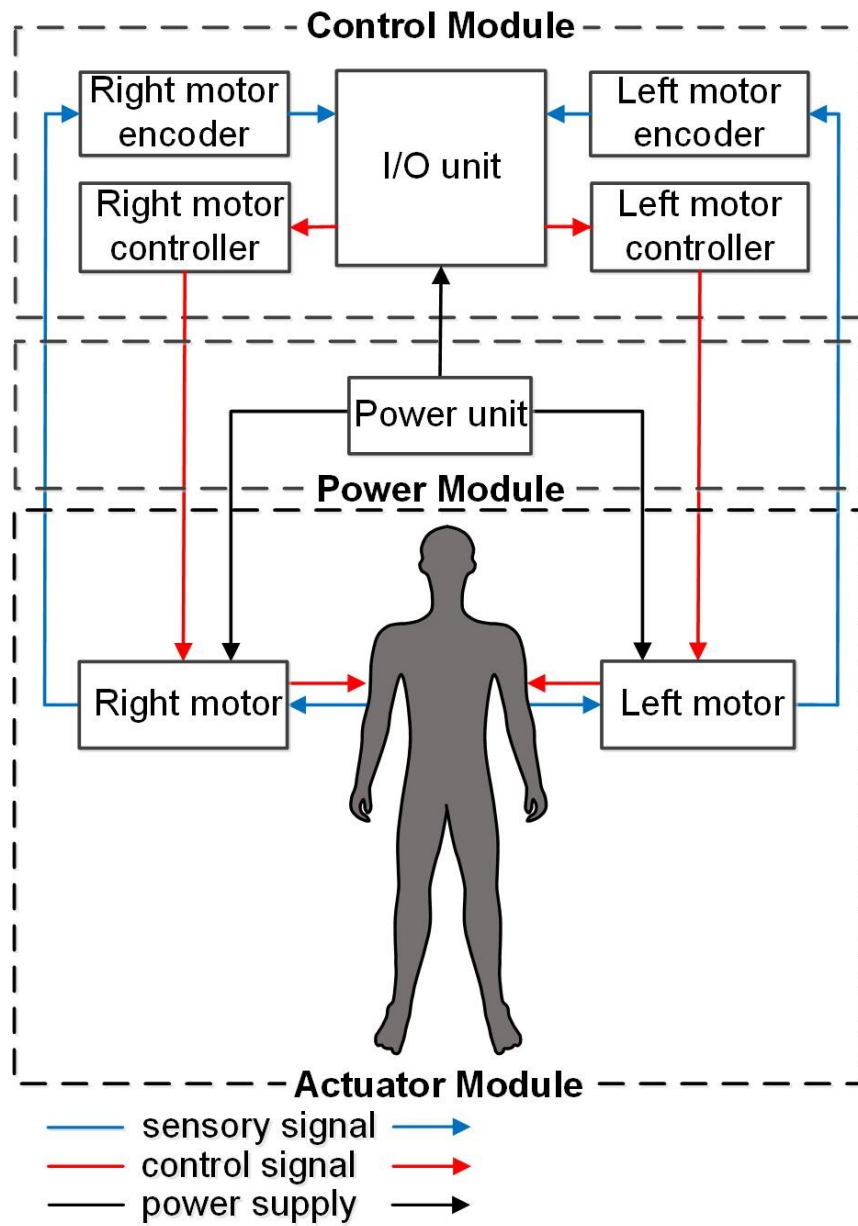

Supplement: Supplementary file 3 [file Image_1.pdf]

**A**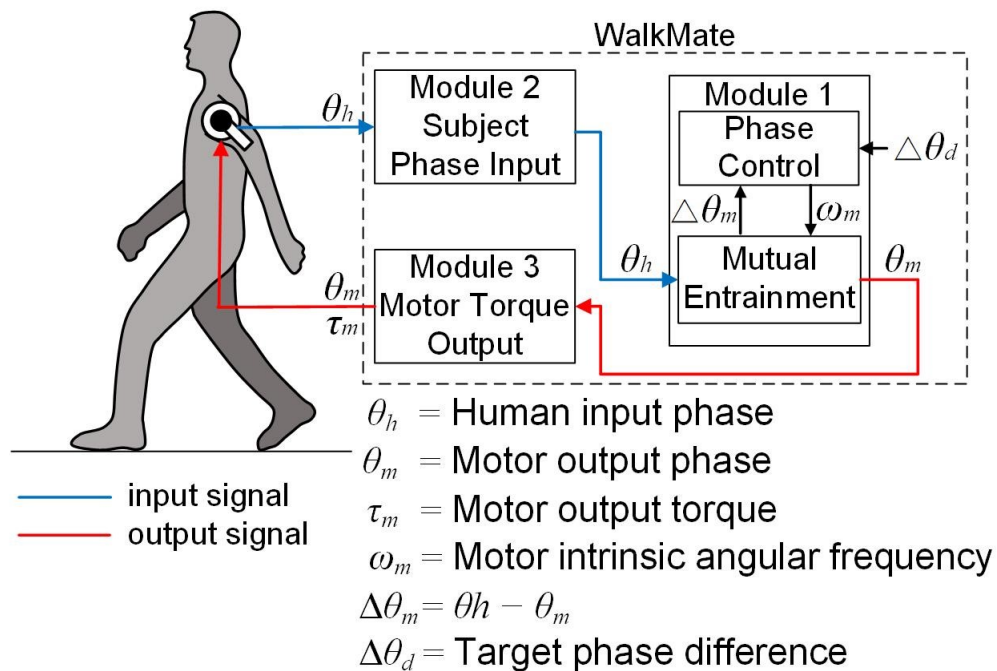**B**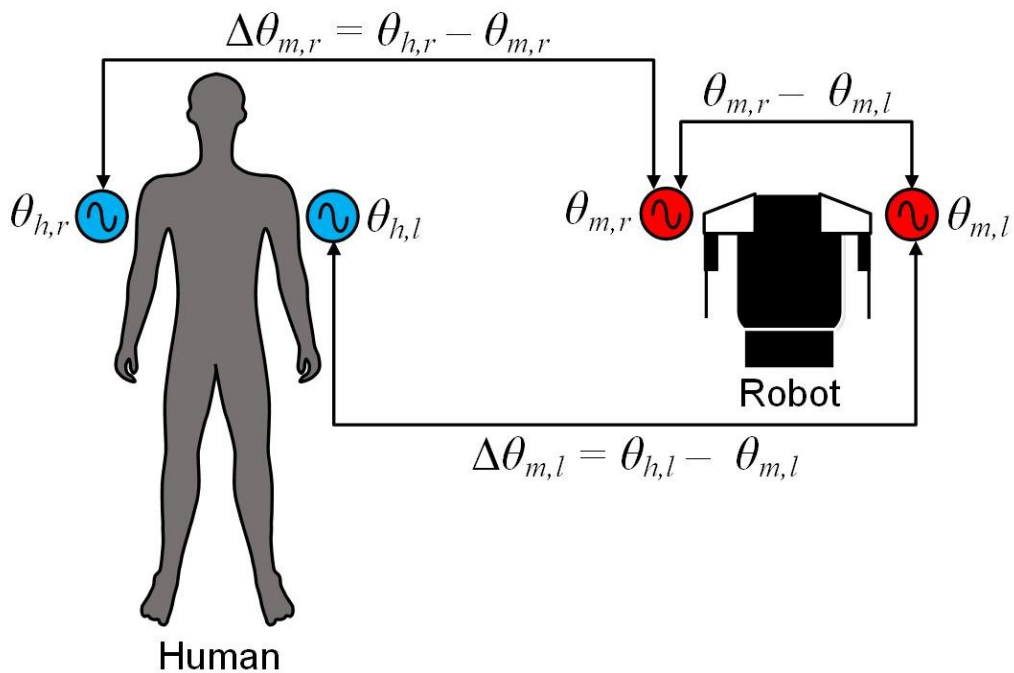**C**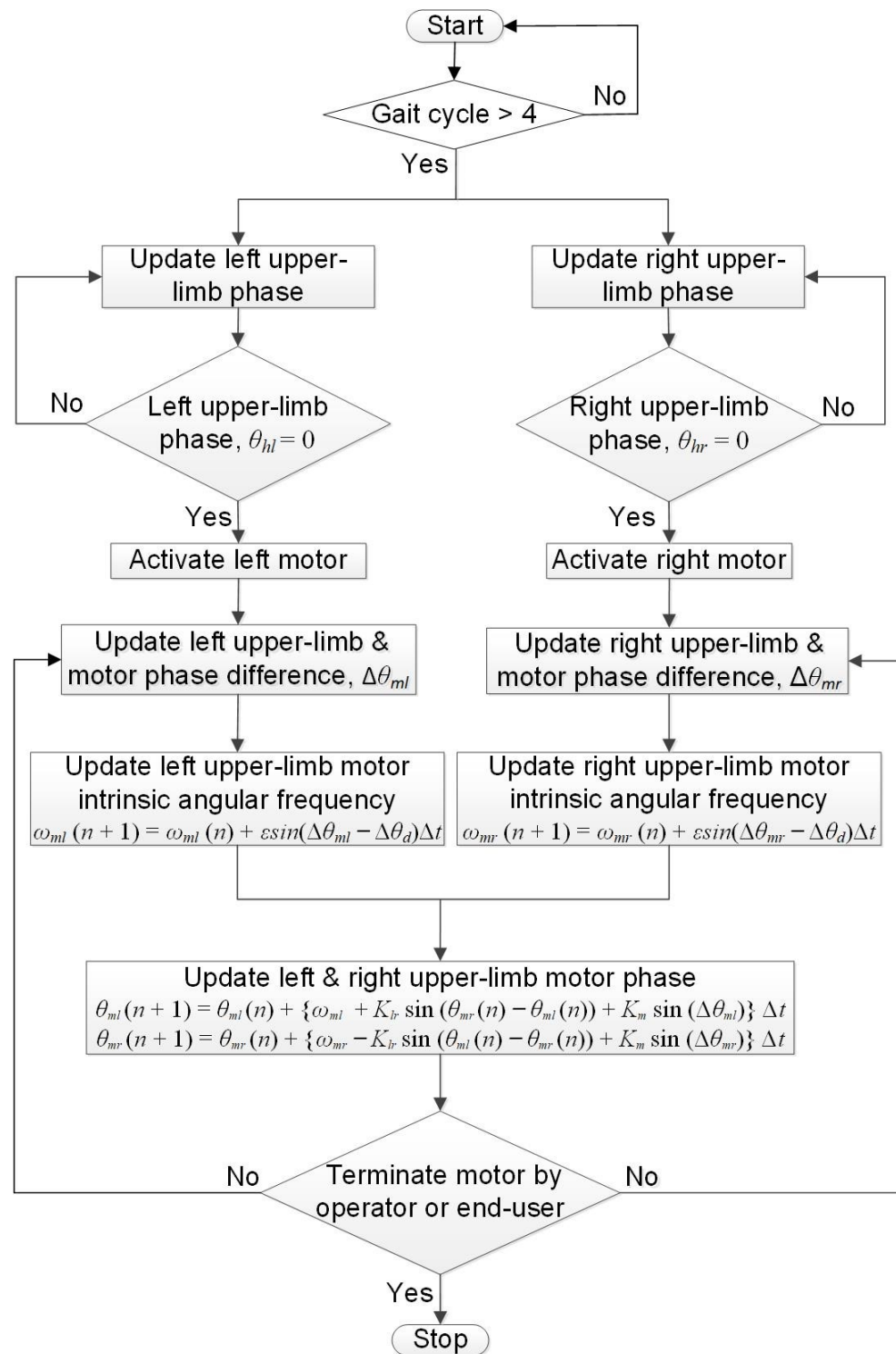

Supplement: Supplementary file 4 [file Image_2.pdf]

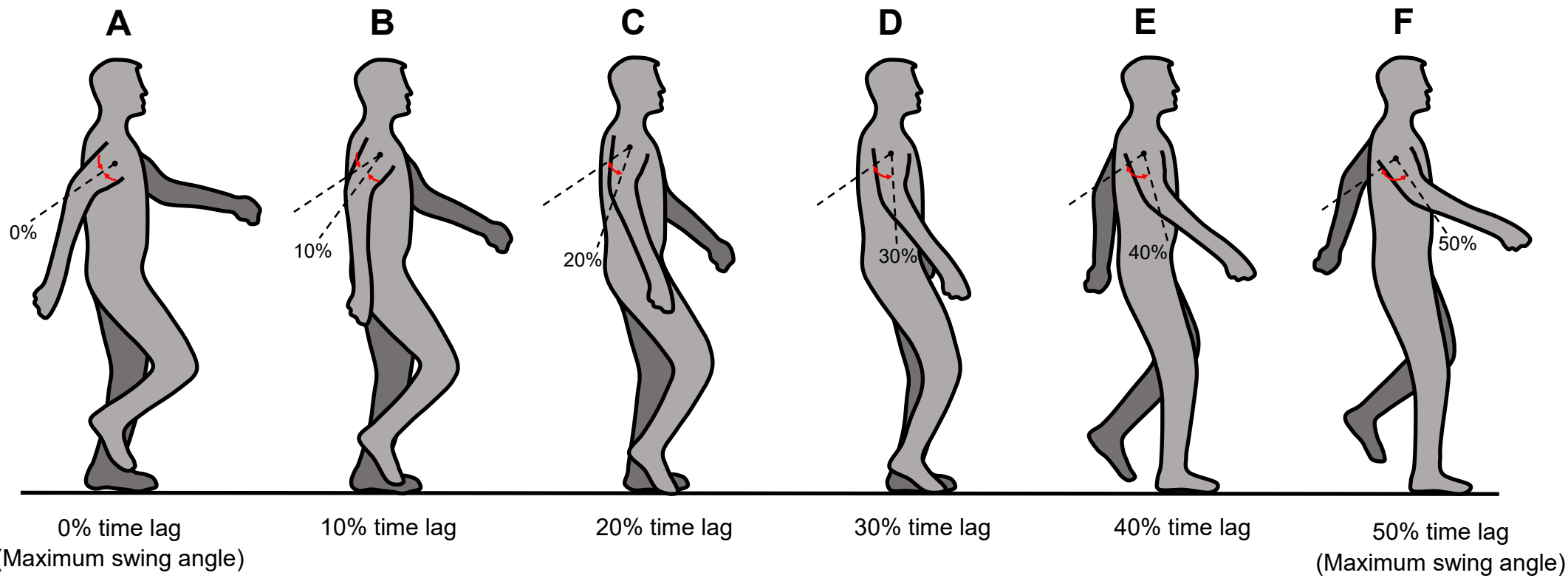

Supplement: Supplementary file 5 [file Image_3.pdf]

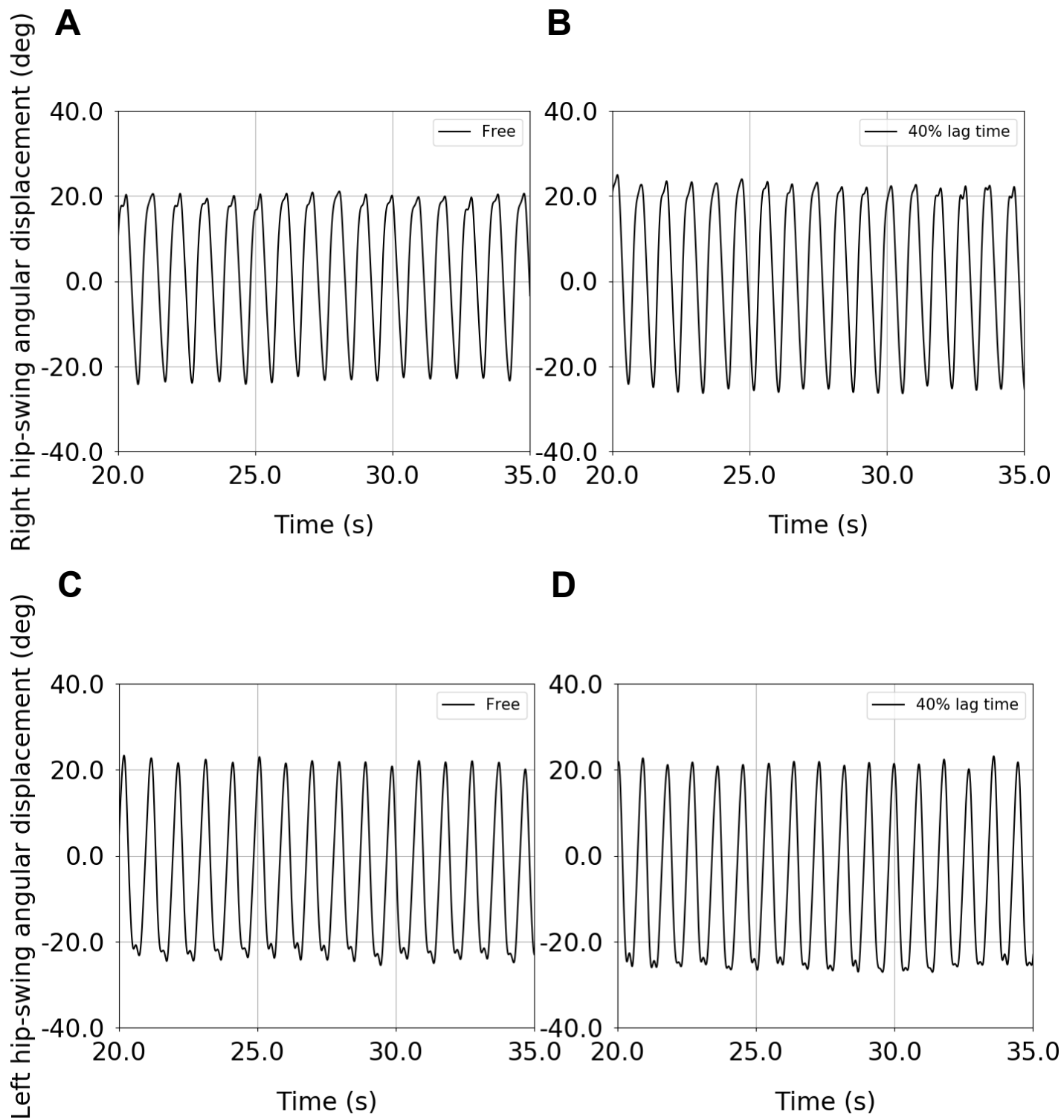

Supplement: Supplementary file 6 [file Image_4.pdf]

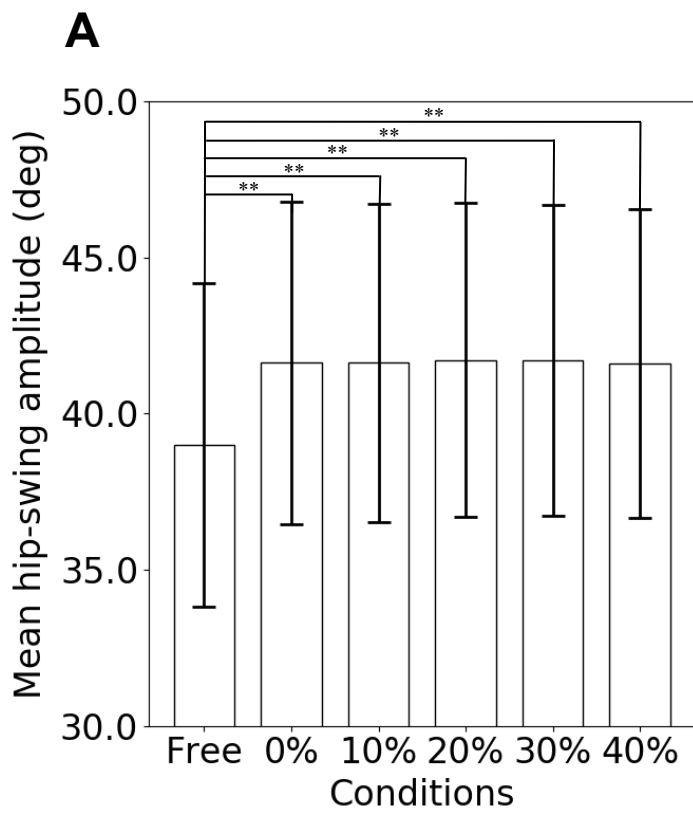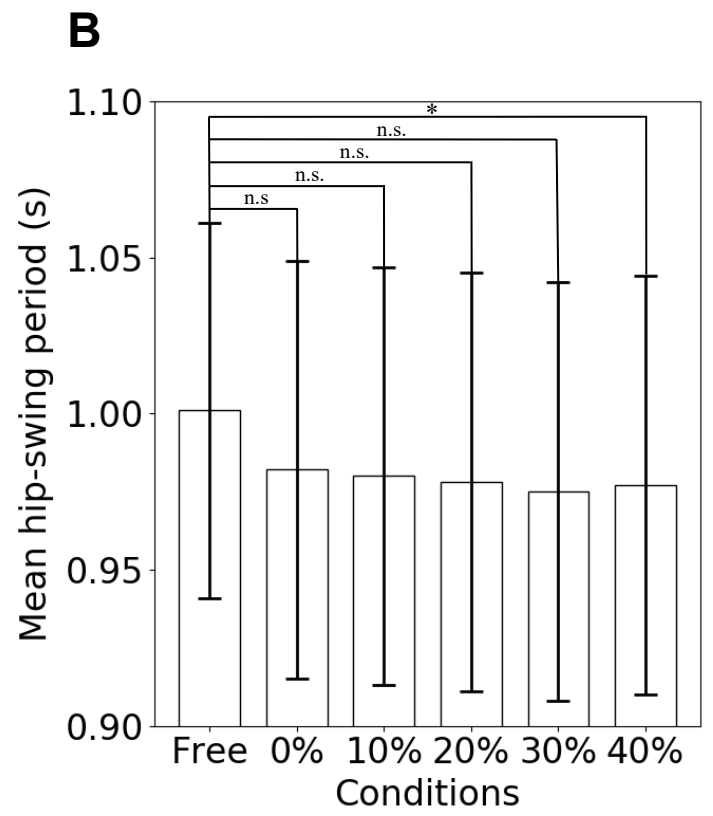

Supplement: Supplementary file 7 [file Image_5.pdf]
